# Tumor Cell Derived Exosomal GOT1 Suppresses Tumor Cell Ferroptosis to Accelerate Pancreatic Cancer Progression by Activating Nrf2/HO-1 Axis via Upregulating CCR2 Expression

**DOI:** 10.3390/cells11233893

**Published:** 2022-12-02

**Authors:** Yao Guo, Taoyu Chen, Xueyi Liang, Shanmiao Gou, Jiongxin Xiong, Jing Cui, Tao Peng

**Affiliations:** Department of Pancreatic Surgery, Union Hospital, Tongji Medical College, Huazhong University of Science and Technology, Wuhan 430022, China

**Keywords:** pancreatic cancer, exosome, GOT1, CCR2, ferroptosis, the Nrf2/HO-1 axis

## Abstract

Recently, evidence has shown that GOT1 expression is upregulated in pancreatic cancer tissues and promotes cancer development, but the specific mechanism remains unclear. We found that GOT1 expression was upregulated in pancreatic cancer cell-derived exosomes. When PANC-1 cells were incubated with exosomes alone or transfected together with si-GOT1, we found that exosomes enhanced cell proliferation, invasion and migration, promoted ferroptosis, and si-GOT1 reversed the effects of exosomes. The results of online bioinformatics database analysis indicated that CCR2 was a potential binding protein of GOT1 and is highly expressed in pancreatic cancer tissues. PANC-1 cells were transfected with pcDNA-CCR2 or si-CCR2, and it was found that pcDNA-CCR2 enhanced cell proliferation, invasion and migration, promoted ferroptosis, and si-CCR2 had an opposite effect. Next, exosome-treated cells were transfected with si-GOT1 alone or together with pcDNA-CCR2, and we found that exosomes promoted CCR2 expression, promoted cell proliferation and invasion, and inhibited ferroptosis, the transfection of si-GOT1 abolished the effect of exosomes, and the transfection of pcDNA-CCR2 again reversed the effect of si-GOT1. Furthermore, when exosome-treated cells were transfected with si-GOT1 alone or co-incubated with Nrf2 activator NK-252, we found that si-GOT1 reversed the promoting effect of exosomes on Nrf2 and HO-1 expression, as well as its inhibitory effect on ferroptosis, but this effect was abrogated by NK-252. In vivo studies showed that knockdown of GOT1 expression inhibited tumor formation compared with tumor tissues formed upon exosome induction, which was mediated by promoting ferroptosis via suppressing the protein expression of GOT1, CCR2, Nrf2 and HO-1 in tumor tissues.

## 1. Introduction

Pancreatic cancer is currently one of the cancers with the highest mortality and lowest survival rate of all cancers. Pancreatic cancer has been reported to have an overall 5-year survival rate of less than 7%, accounting for the fourth in tumor related lethality, and it is expected to reach the second position by 2030 [1]. Although radical surgery increases the 5-year survival rate of pancreatic cancer to 15–20%, its long-term survival rate has not improved significantly [2]. Therefore, novel therapeutic strategies for pancreatic cancer need to be developed urgently.

The biological characteristics of tumor cells are modulated by the microenvironment in which they reside, in which s are important mediators of “communication” between cells, and can transport molecules such as proteins, mRNAs, or miRNAs to recipient cells, thereby modulating metastasis and therapeutic resistance. It has been confirmed that stromal cells weaving complex communication networks with breast cancer cells via exosomes enhances therapeutic resistance [3]. In addition, miR-208a reached lung cancer cells to target p21 via exosomal trafficking, thus affecting lung cancer proliferation and radiotherapy sensitivity [4]. In a related study of pancreatic cancer, Wang et al. showed that plasma derived exosomal miR-19b expression levels in pancreatic cancer patients were significantly lower than those in other pancreatic tumor patients, chronic pancreatitis patients, and healthy volunteers, suggesting that plasma derived exosomal miR-19b may be a promising diagnostic marker for pancreatic cancer [5]. Another study found increased expression of miR-155 in PDAC cells chronically exposed to gemcitabine, and miR-155 was able to promote exosome secretion, which in turn resulted in the resistance of tumor cells to gemcitabine [6].

For decades, an increasing number of studies have demonstrated that cellular metabolism is closely related to malignant tumorigenesis. However, unlike normal cells, cancer cells reprogram metabolism to meet the substantial material and energy demands of their rapid proliferation. Many metabolic pathways appear reprogrammed in cancer, including glycolysis, the TCA cycle, glutaminolysis, the electron transport chain, and the pentose phosphate pathway [7,8]. Since the discovery of the Warburg effect, more and more studies have proved that the metabolism of cancer cells plays a crucial role in cancer survival and growth, and glutamine plays a more important role in cancer metabolism than previously thought. It is well documented that several human pancreatic ductal adenocarcinoma (PDAC) cell lines rely on the Kirsten ratsarcoma viral oncogene homolog regulated non canonical glutamine (Gln) metabolic pathway to promote tumor cell proliferation and growth [9]. In PDAC cells, KRAS specific dependent downregulation of glutamate dehydrogenase (GLUD1) and the upregulation of aspartate aminotransferase 1 (GOT1) promotes the derivation of glutamine into aspartate, which is converted into oxaloacetate (OAA) in the cytoplasm by GOT1, followed by a series of transformations into pyruvate, ultimately promoting the production of NADPH for maintaining redox balance [9]. In addition, the proliferation of PDAC cells is also inhibited upon selective inhibition of GOT1 expression in tumor cells, and these phenomena suggest that targeting GOT1 may be a novel approach for the treatment of pancreatic ductal carcinoma [10]. A recent publication showed that aspartate aminotransferases (GOTS) were identified as key metabolic enzymes for human PDAC. Yang et al. found that acetylation of GOT2 could promote ATP production and stimulate NADPH generation to inhibit ROS generation [11]. These studies collectively establish that GOTS play an important role in redox regulation in human pancreatic cancer, laying the foundation for the treatment of cancer by targeting GOTS.

The tumor microenvironment is a key factor influencing tumor growth, spread, and metastasis. As an important and widely studied chemokine in the tumor microenvironment, monocytechemoattractant protein-1 (MCP-1/CCL2) is one of the important members of the CC subfamily of chemokines with two forms, autocrine or paracrine. CCL2 exerts its biological effects mainly through binding to its receptor C-C motif chemokine receptor-2 (CCR2), and numerous studies have shown that the chemokine CCL2 and its receptor CCR2 are highly expressed in a variety of malignancies. Zhuang et al. found that CCL2 could activate the hedgehog signaling pathway of HCC cells through the CCL2/CCR2 molecular axis, and up regulate the expression of snail and vimentin, as well as down regulate E-cadherin expression to promote HCC cell invasion and epithelial mesenchymal transition [12]. Li et al. showed that CCR2 expression was abnormally elevated in HCC tissues and significantly correlated with tumor volume, metastasis and clinical stage, and that the high expression of CCR2 in HCC tissues was significantly associated with the poor prognosis of patients [13]. Independently, CCL2 was reported to recruit monocytes and reduce CD8^+^ T cell infiltration in pancreatic tumors, and CCL2 inhibition and monocyte neutralisation increased the sensitivity of PDAC to immune checkpoint blockade [14]. Currently, although numerous studies have confirmed the cancer promoting role of CCR2 in multiple tumors, its role in pancreatic cancer progression is not well understood. Therefore, investigating the role and possible mechanisms of CCR2 in pancreatic cancer progression will be of great importance to unearth new therapies against pancreatic cancer.

## 2. Materials and Methods

### 2.1. Cell Culture

The human pancreatic cancer cell lines AsPC-1 (RRID: CVCL_0152), BxPC-3 (RRID: CVCL_0186), PANC-1 (RRID: CVCL_0480) and SW1990 (RRID: CVCL_1723), and the normal human pancreatic ductal epithelial cell line HPDE were purchased from American Type Culture Collection (ATCC, Manassas, VA, USA). The cells were incubated in medium containing 10% FBS and 100 U/mL penicillin and 100 μg/mL streptomycin (Sigma, St. Louis, MO, USA) in RPMI 1640 medium (Gibco, Rockville, MD, USA) at 5% CO_2_ at 37 °C. The pcDNA-GOT1, pcDNA-CCR2 and their negative control (vector) were purchased from RiboBio Co., Ltd. (Guangzhou, China) and transfected using RiboBio Transfection Kit (RiboBio Co., Ltd.). Small interfering RNA against GOT1 (si-GOT1), CCR2 (si-CCR2) and their negative control (scramble) were purchased from Santa Cruz Biotechnology (Santa Cruz, CA, USA). All transfection reagent transfected into cells using Lipofectamine 3000 Transfection Reagent (Invitrogen, Carlsbad, CA, USA) according to the manufacturer’s instructions. The cell lines used in this study have been STR authenticated and determined to be contamination free. The absence of mycoplasma was checked every 6 months (MycoAlert™ mycoplasma detection kit, Lonza, Ozyme, Saint-Cyr L’Ecole, France).

### 2.2. Animals

Adult nude mice (weight 20–22 g, Wuhan Experimental Animal Center, China) were housed in a specific pathogen-free environment under the condition of 12-h light/12-h dark cycle, free access to food and water, and acclimatized to their surroundings for three days. These nude mice were randomly divided into two groups (*n* = 8 per group) including scramble group (exosomes were secreted by PANC-1 cells that transfected with scramble) and si-GOT1 group (exosomes were secreted by PANC-1 cells that transfected with si-GOT1). The exosomes (250 μL) secreted by PANC-1 cells transfected with scramble or si-GOT1, were subcutaneously injected into the armpits of nude mice. The tumor volume was closely observed and measured on days 8, 11, 14, 17, 20 and 23 after injection. On day 23, nude mice were euthanized, and tumor tissues were collected subjected to subsequent studies. All experiments were conducted in accordance with the Animal Ethics Committee of Union Hospital, Tongji Medical College, Huazhong University of Science and Technology.

### 2.3. Exosome Extraction and Identification

The supernatant of cell culture medium was collected, and the cell components and dead cells were removed by low-speed centrifugation (300× *g* × 10 min, 2000× *g* × 10 min) at 4 °C. The supernatant containing exosomes was retained and the cell debris was removed by high-speed centrifugation (10,000× *g* × 70 min). The supernatant containing extracellular vesicles was retained and the exosomes were precipitated by ultracentrifugation (100,000× *g* × 70 min). Appropriate amount of PBS was taken to resuspend the extracellular vesicle precipitation, and then ultracentrifuged again (100,000× *g* × 70 min) to eliminate contaminated proteins. The precipitation was collected and divided, and stored at −80 °C for future use. The size and concentration of the exosomes were analyzed by a nanoparticle tracking analysis (NTA) instrument (ZetaView, Particle Metrix, Meerbusch, Germany). The morphology of exosomes was observed using a transmission electron microscopy (TEM, StarJoy, Japan; JSM-7800F).

### 2.4. RT-qPCR

Total RNA was isolated from tumor tissues by using TRIzol (Invitrogen, Carlsbad, CA, USA). Single-stranded cDNA was synthesized with the PrimeScript Reagent Kit (Promega, USA). Real-time qPCR was conducted by using SYBR Premix Ex TaqTM Kit (Applied Biosystems, Foster City, CA, USA). The reaction was run in ABI7500 Real-time PCR system (Applied Biosystems, Carlsbad, CA, USA). GAPDH was used as an endogenous control. The PCR cycling conditions consisted of: 95 °C for 3 min; then, 35 cycle amplification for 20 s at 95 °C, 30 s at 55 °C, 15 s at 72 °C; followed by 1 min at 72 °C. The primers used in this study were synthesized by Sangon Biotech (Shanghai, China). The expression level was normalized by using the 2^−ΔΔCt^ method.

### 2.5. Western Blotting

Briefly, the cells were lysed for 20 min on ice in ice-cold lysis buffer (Roche). The lysates were centrifuged at 12,000× *g* for 20 min at 4 °C to obtain a clear lysate. The protein content of each sample was determined using the BCA Protein Assay Kit (Thermo Scientific). Then, equal amounts of proteins(15 μg/lane) were separated on a 12% sodium dodecyl sulfate polyacrylamide gel electrophoresis (SDS-PAGE) and transferred to polyvinylidenedifluoride (PVDF) membranes (Bio-Rad, Hercules, CA, USA).The membranes were blocked in 5% (*w*/*v*) nonfat dry milk in TBST (Tris-buffered saline-0.1% Tween) at 25 °C for 3 h and then incubated with the following primary antibodies: GAPDH antibody (1:1000, Abcam, ab8245), CD63 antibody (1:1000, Abcam, ab217345), TSG101 antibody (1:2000, Abcam, ab125011), Alix antibody (1:8000, Abcam, ab88388), GOT1 antibody (1:500, Abcam, ab85857), GXP4 antibody (1:800, Abcam, ab75810), CCR2 antibody (1:900, Abcam, ab203128), Nrf2 antibody (1:1000, Abcam, ab92946) and HO-1 antibody (1:2000, Abcam, ab52947). Then, the membranes were incubated with horseradish peroxidase (HRP)-conjugated goat anti-rabbit IgG (1:10,000, Abcam, ab205718) for 1 h. The protein bands were visualized by using the Enhanced chemiluminescence reagents (Millipore, MA, USA). The expression of relative protein was obtained by the gray value ratio of the target protein to the internal reference GAPDH and analyzed with the ImageJ software (National Institutes of Health, Bethesda, MA, USA).

### 2.6. Immunohistochemical Assay

Briefly, tumor tissue sections were pretreated with trypsin (0.05%) for 10 min and then treated with 3% (*v*/*v*) H_2_O_2_. Sections were then blocked with 10% goat serum for 1 h at room temperature. After washing with PBS, anti-GOT1 antibody, anti-CCR2, anti-Nrf2 or anti-HO-1 (1:50 dilution) was applied to the sections, and the sections were incubated overnight at 4 °C. Sections were then washed with PBS for 15 min and incubated with biotinylated secondary antibodies by using the Histostain Plus Kit (Invitrogen, Carlsbad, CA, USA). Sections were washed and incubated with 3, 30-diaminobenzidine (DAB) substrate for 2 min.

### 2.7. MTT Assay

Cell viability was determined by MTT kits (Dojindo, Kumamoto, Japan). The PANC-1 cells were seeded in a 96-well plate at 1.5 × 10^4^ cells/well. At 24, 48 and 72 h after transfection, the supernatant was discarded and 20 μL of MTT solution was added to each well. After incubating at 37 °C for 4 h, the absorbance at 490 nm was measured and recorded. Each experimental procedure was processed at least three times.

### 2.8. Transwell Cell Invasion Assay

Cells were seeded into the upper chamber of Transwell chambers (8.0 μm pore size; Millipore Corporation, Boston, MA, USA) coated with Matrigel (BD Bioscience, Franklin lakes, NJ, USA). The complete medium was added into the lower chamber. After incubation at 37 °C for 48 h, cells on the upper chamber were removed with cotton swabs, while cells on the lower champer were fixed with 70% ethanol and stained with 0.1% crystal violet. The invasive cells were counted under a light microscope (Olympus, Tokyo, Japan).

### 2.9. Cell Colony Formation Assay

PANC-1 and SW1990 cells were seeded in 6-well plates. Media were renewed every 3 days during culture. Two weeks later, cell supernatant was removed, and proliferative colonies were incubated with paraformaldehyde (Sigma) and crystal violet (Sigma), respectively. Cell colony-forming ability was illustrated via counting cell numbers. A colony was deemed when cell numbers >50.

### 2.10. Wound-Healing Assay

PANC-1 and SW1990 cells were cultured in 6-well plates until their confluence reached about 100%. Then, cell wounds were created and cells were washed using phosphate buffer solution (PBS; Thermo Fisher, Waltham, MA, USA). Cells were continued to be cultured in serum-free media for 24 h. Finally, cell migratory ability was determined via calculating the width of wounds under microscope (Nikon) with a 100(×) magnification.

### 2.11. Co-IP Assay

After 48 h of transfection, cells were washed twice with cold PBS, and then RIPA lysis buffer was added for lysis. The supernatant collected by centrifugation was incubated with corresponding antibodies at 4 °C overnight. Then, we added 100 μL of Protein A agarose beads to capture the antigen-antibody complex, and slowly shook the mixture at 4 °C overnight. The agarose beads-antigen-antibody complex was collected by instantaneous centrifugation, and washed with ice-cold PBS. Then, the complex was boiled with protein loading buffer to free the antigen, antibody and beads. After centrifugation, the supernatant was taken for electrophoresis to detect the expression of the interaction protein.

### 2.12. ROS Content Detection

Cells were plated at 1 × 10^4^ density seeded in culture flasks. ROS kit was used to measure ROS levels according to the manufacturer’s instructions. Dichloro-dihydro-fluorescein diacetate (DCFH-DA; 10 μM) was added to the cells and incubated for 20 min at 37 °C. The cells were then digested and suspended. The cell suspensions were centrifuged at 1000× *g* for 10 min and washed twice with phosphate-buffered saline (PBS). The cells were collected after centrifugation for fluorescence detection. Flow cytometry was used to measure fluorescence intensity. The positive area of DCFH-DA was ROS fluorescence intensity.

### 2.13. Determination of MDA and Fe^2+^ Contents

The secretion of MDA in PANC-1 cell supernatant was detected by ELISA kits (Sigma) following the manufacturer’s instructions. Similarly, the levels of Fe^2+^ were detected by Iron Colorimetric Assay Kits (Sigma).

### 2.14. Statistical Analysis

All statistical analyses were performed by using the SPSS software (ver. 21.0; SPSS, Chicago, IL, USA). The quantitative data derived from three independent experiments were expressed as mean ± SD. Comparisons between two groups were made by the Student’s *t*-test. Data between multiple groups were performed with one-way analysis of variance (ANOVA) followed by post hoc analysis with LSD test. *p* < 0.05 was considered statistically significant.

## 3. Results

### 3.1. GOT1 Expression Was Upregulated in Pancreatic Cancer Tissues and Cell Lines

**Figure 1:**
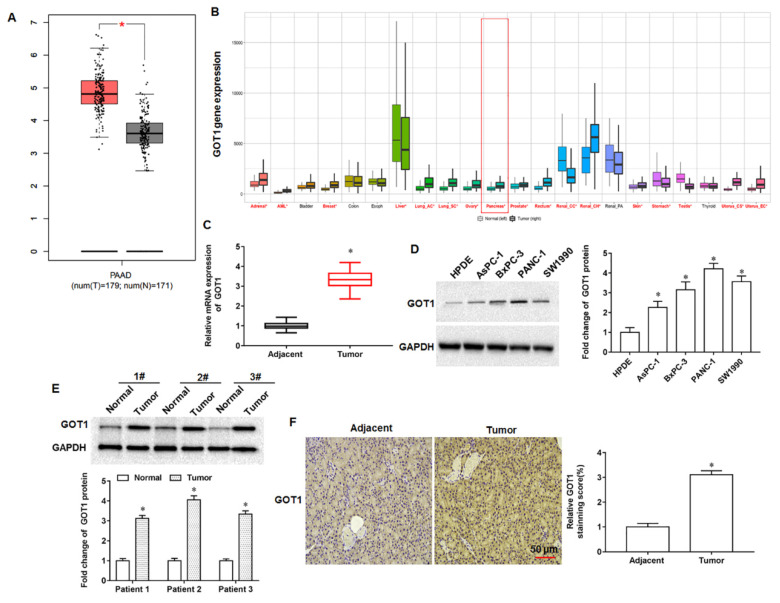
Expression of GOT1 in pancreatic cancer tissue samples. (**A**,**B**) The expression levels of GOT1 were analyzed by an online bioinformatics database (http://gepia.cancer-pku.cn/index.html; accessed on 13 May 2019; https://tnmplot.com/analysis/; accessed on 13 May 2019). (**C**) The relative mRNA expression of GOT1 was detected by RT-qPCR (Samples were obtained from tumor tissues and adjacent tissues from 30 tumor patients). (**D**) Western blotting was used to detect GOT1 protein expression in pancreatic cancer cell lines AsPC1, BxPC-3, PANC-1 and SW1990, and the normal human pancreatic ductal epithelial cell line HPDE. (**E**) Western blotting was used to detected GOT1 protein expression in tumor tissues of three patients. (**F**) Representative images of immunohistochemical staining of GOT1 in tumor tissues. * *p* < 0.01.

We found abnormally high expression of GOT1 in pancreatic cancer progression by bioinformatics database analysis. (http://gepia.cancer-pku.cn/index.html, accessed on 13 May 2019; Figure 1A; https://tnmplot.com/analysis/, accessed on 13 May 2019; Figure 1B). Next, we examined the collected tumor tissues and adjacent tissues from 30 pancreatic cancer patients and found that GOT1 expression was significantly elevated in tumor tissues compared with adjacent tissues (Figure 1C). Furthermore, the expression of GOT1 was upregulated in pancreatic cancer cell lines AsPC1, BxPC-3, PANC-1 and SW1990 compared with normal human pancreatic ductal epithelial cell line HPDE (Figure 1D). In addition, we selected three pairs of tumor tissues and adjacent tissues to examine, and the results of Western blotting indicated that GOT1 expression was increased in tumor tissues compared with adjacent tissues (Figure 1E). Through immunohistochemical staining sections of tumor tissues and adjacent tissues, we observed that the number of GOT1 positive cells in tumor tissues was more than that in adjacent tissues (Figure 1F). The above results showed that GOT1 expression was upregulated in pancreatic cancer tissues and cell lines, suggesting that GOT1 may be involved in pancreatic cancer progression.

### 3.2. Identification of Exosomes from PANC-1 and SW1990 Cells

**Figure 2:**
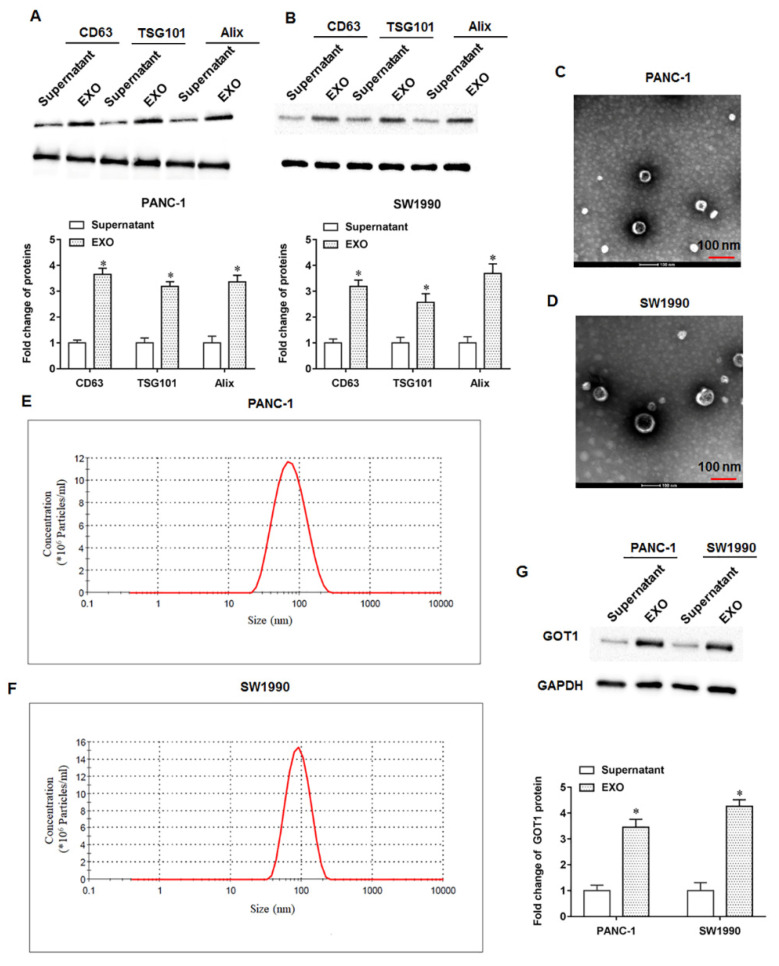
Identification of exosomes from PANC-1 and SW1990 cells. The culture medium supernatants of PANC-1 and SW1990 cells were collected, and the pellets were obtained by ultracentrifugation and extraction of tumor cell-derived exosomes. (**A**,**B**) The expression levels of exosome marker proteins (CD63, TSG101 and Alix) in the supernatant (centrifugation) and pellet were detected by Western blotting. (**C**,**D**) Representative images of PANC-1 and SW1990 cell-derived exosome vesicles under transmission electron microscopy field of view. Scale bar: 100 nm. (**E**,**F**) The particle size of exosomes was measured by NTA. (**G**) The protein expression levels of GOT1 in PANC-1 and SW1990 cell-derived exosomes were detected by Western blotting. * *p* < 0.01.

We collected the medium supernatant of PANC-1 and SW1990 cells, respectively, and performed ultracentrifugation to obtain a pellet. Western blotting showed that the exosomal marker proteins CD63, TSG101 and Alix were expressed at significantly higher levels in the pellet than in the cell supernatant (Figure 2A,B). Next, we characterized the PANC-1 and SW1990 cell-derived exosomes by TEM, and we observed that the exosome vesicles appeared as round particles under the electron microscope field (Figure 2C,D). Typically, the exosomes appeared theophyllo like with a slightly brighter bright circle at the edge. If the structure is a ball shape without a membrane structure, it is most likely a lipoprotein particle or aspiration protein. The particle size of exosomes was measured by NTA and the results were shown in Figure 2E,F. Furthermore, Western blot results indicated the high expression of GOT1 protein in PANC-1 and SW1990 cell-derived exosomes compared with cell culture medium supernatants (Figure 2G). The above results indicate that GOT1 is upregulated in exosomes secreted by pancreatic cancer cells, suggesting that GOT1 may participate in pancreatic cancer progression through the tumor microenvironment.

### 3.3. Exosomal GOT1 Promoted Tumor Cell Growth and Inhibited Ferroptosis

**Figure 3:**
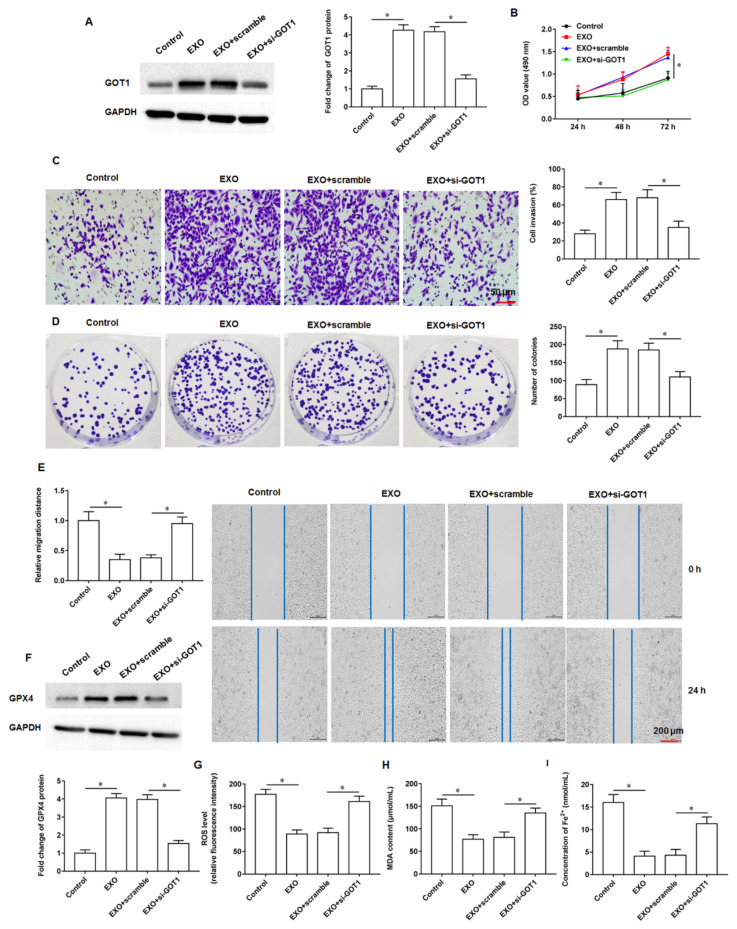
Effect of exosomal GOT1. The 150 μL of exosomes were added to PANC-1 cells alone or transfected together with si-GOT1. (**A**) Western blotting was used to detect GOT1 protein expression. (**B**) MTT assay was used to analyze cell proliferation. (**C**) Transwell invasion assay was used to detect cell invasion. (**D**) Cell colony formation assay was used to analyze cell proliferation. (**E**): Wound healing assay was used to detect cell migration. (**F**) The GPX4 protein expression was detected by Western blotting. (**G**) The ROS content was detected by flow cytometry. (**H**,**I**) MDA and Fe^2+^ contents were detected by using ELISA kits or Iron Colorimetric Assay Kits, respectively. * *p* < 0.01.

In results 1 and 2, we confirmed that GOT1 was abnormally highly expressed in pancreatic cancer tissues and cells and that GOT1 was abundantly enriched in PANC-1 and SW1990 cell-derived exosomes, so the effects of exosomal GOT1 on tumor cells warrant further exploration. Here, the 150 μL of exosomes were added to PANC-1 cells alone or transfected together with si-GOT1. We found that GOT1 protein expression was increased (increased approximately 4-fold) in cells incubated with exosomes alone, and transfection of si-GOT1 reversed the promoting effect of exosomes on GOT1 protein expression (Figure 3A). MTT assay results indicated that exosomes promoted cell proliferation (enhanced approximately 2.5-fold) and knockdown of GOT1 inhibited cell proliferation (Figure 3B). In addition, we observed that cells incubated with exosomes alone had significantly enhanced abilities of invasion (enhanced approximately 2.1-fold, Figure 3C), clonogenicity (enhanced approximately 2.4-fold, Figure 3D) and migration (enhanced approximately 3.3-fold, Figure 3E), and the transfection of si-GOT1 partially abolished the effects of exosomes. Next, we found that the expression of GPX4 protein was increased (increased approximately 3.8-fold, Figure 3F) and the content of ROS (increased approximately 2-fold, Figure 3G), MDA (increased approximately 2.2-fold, Figure 3H) and Fe^2+^ (increased approximately 3-fold, Figure 3I) were decreased in cells treated with exosomes, suggesting that exosomes inhibit ferroptosis in PANC-1 cells, and this inhibitory effect was counteracted after knockdown of GOT1. In addition, SW1990 cells were treated with SW1990 cell-derived exosomes alone or in-combination with si-GOT1. It was found that exosomes promoted GOT1 protein expression (increased approximately 4.2-fold, Supplementary Figure S1A), enhanced cell invasion (enhanced approximately 1.9-fold, Supplementary Figure S1B) and clone formation (enhanced approximately 2.3-fold, Supplementary Figure S1C), and inhibited ferroptosis (Figure 1D–F), while si-GOT1 reversed the effect of exosomes. The above results indicate that exosomes enriched with GOT1 promote pancreatic cancer cell proliferation, invasion, clonogenicity and migration, and inhibit cellular ferroptosis.

### 3.4. CCR2 Expression Was Upregulated in Pancreatic Cancer Tissues and Cell Lines

**Figure 4:**
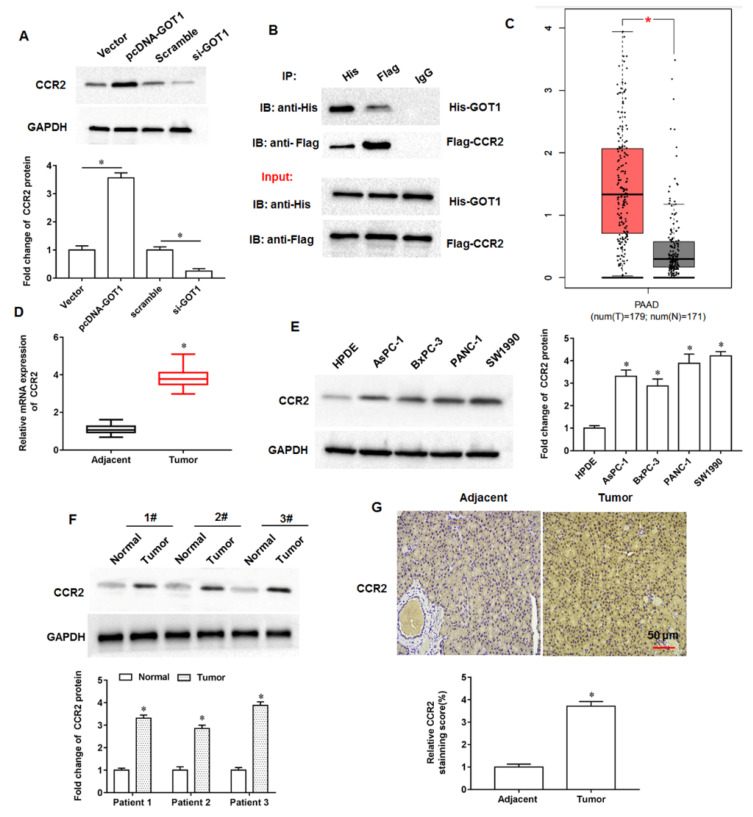
Expression of CCR2 in pancreatic cancer tissue samples. (**A**) The cells were transfected with pcDNA-GOT1 and si-GOT1, and CCR2 protein expression was detected by Western blotting. (**B**) The binding of GOT1 and CCR2 was confirmed by Co-immunoprecipitation assay. (**C**) The expression levels of CCR2 were analyzed by an online bioinformatics database (http://gepia.cancer-pku.cn/index.html; accessed on 25 May 2020). (**D**) The relative mRNA expression of CCR2 was detected by RT-qPCR (Samples were obtained from tumor tissues and adjacent tissues from 30 tumor patients). (**E**) Western blotting was used to detect CCR2 protein expression in pancreatic cancer cell lines AsPC1, BxPC-3, PANC-1 and SW1990, and the normal human pancreatic ductal epithelial cell line HPDE. (**F**) Western blotting was used to detected CCR2 protein expression in tumor tissues of three patients. (**G**) Representative images of immunohistochemical staining of CCR2 in tumor tissues. * *p* < 0.01.

The results of online bioinformatics analysis indicated that CCR2 protein might be a potential regulatory target of GOT1 (https://thebiogrid.org; accessed on 25 May 2020). Next, we found that the overexpression of GOT1 promoted CCR2 protein expression and knockdown of GOT1 inhibited CCR2 expression (Figure 4A), and the binding relationship between GOT1 and CCR2 was verified by co-immunoprecipitation (Figure 4B).

We found an abnormally high expression of CCR2 in pancreatic cancer progression by bioinformatics database analysis. (http://gepia.cancer-pku.cn/index.html, accessed on 25 May 2020; Figure 4C). Next, we examined the collected tumor tissues and adjacent tissues from 30 pancreatic cancer patients and found that CCR2 expression was significantly elevated in tumor tissues compared with adjacent tissues (Figure 4D). Furthermore, the expression of CCR2 was upregulated in pancreatic cancer cell lines AsPC1, BxPC-3, PANC-1 and SW1990 compared with normal human pancreatic ductal epithelial cell line HPDE (Figure 4E). In addition, we selected three pairs of tumor tissues and adjacent tissues to examine, and the results of Western blotting indicated that CCR2 expression was increased in tumor tissues compared with adjacent tissues (Figure 4F). Through immunohistochemical staining sections of tumor tissues and adjacent tissues, we observed that the number of CCR2 positive cells in tumor tissues was more than that in adjacent tissues. (Figure 4G). The above results indicate that CCR2 is a potential binding protein of GOT1 and that CCR2 is upregulated in pancreatic cancer tissues and cell lines, suggesting that CCR2 may be involved in pancreatic cancer progression.

### 3.5. CCR2 Promoted Tumor Cell Growth and Inhibited Ferroptosis

**Figure 5:**
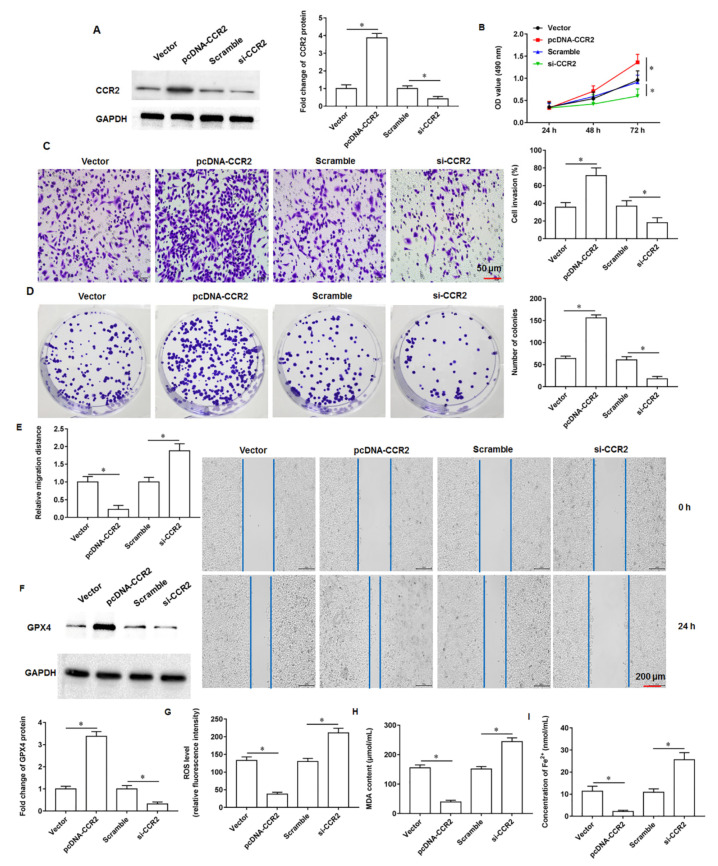
Effect of CCR2. The PANC-1 cells were transfected with pcDNA-CCR2 or si-CCR2. (**A**) Western blotting was used to detect CCR2 protein expression. (**B**) MTT assay was used to analyze cell proliferation. (**C**) Transwell invasion assay was used to detect cell invasion. (**D**) Cell colony formation assay was used to analyze cell proliferation. (**E**) Wound healing assay was used to detect cell migration. (**F**) The GPX4 protein expression was detected by Western blotting. (**G**) The ROS content was detected by flow cytometry. (**H**,**I**) MDA and Fe^2+^ contents were detected by using ELISA kits or Iron Colorimetric Assay Kits, respectively. * *p* < 0.01.

The pcDNA-CCR2 or si-CCR2 were transfected into PANC-1 cells, respectively. The PANC-1 cells were transfected with pcDNA-CCR2 or si-CCR2, respectively. We found that the overexpression of CCR2 promoted CCR2 protein expression and knockdown of CCR2 inhibited CCR2 expression, and we found that pcDNA-CCR2 promoted CCR2 protein expression and si-CCR2 inhibited CCR2 protein expression (Figure 5A). MTT assay results indicated that pcDNA-CCR2 promoted cell proliferation and knockdown of CCR2 inhibited cell proliferation (Figure 5B). In addition, we observed that cells transfected with pcDNA-CCR2 had significantly enhanced abilities of invasion (Figure 5C), clonogenicity (Figure 5D) and migration (Figure 5E), and the transfection of si-CCR2 had the opposite effect. Next, we found that the expression of GPX4 protein was increased (Figure 5F) and the content of ROS (Figure 5G), MDA (Figure 5H) and Fe^2+^ (Figure 5I) were decreased in cells transfected with pcDNA-CCR2, suggesting that pcDNA-CCR2 inhibit ferroptosis in PANC-1 cells. The above results indicate that CCR2 promote pancreatic cancer cell proliferation, invasion, clonogenicity and migration, and inhibit cellular ferroptosis.

### 3.6. Exosomal GOT1 Inhibited Ferroptosis by Upregulating CCR2 Expression

**Figure 6:**
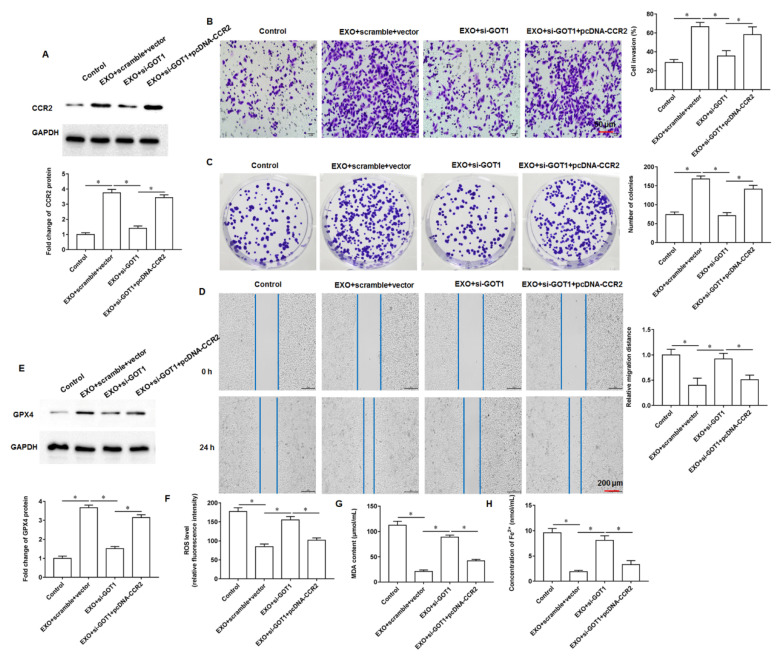
Exosomal GOT1 upregulates CCR2 expression. The exosome (150 μL)-incubated PANC-1 cells were transfected with si-GOT1 alone or together with pcDNA-CCR2. (**A**) Western blotting was used to detect CCR2 protein expression. (**B**) Transwell invasion assay was used to detect cell invasion. (**C**) Cell colony formation assay was used to analyze cell proliferation. (**D**) Wound healing assay was used to detect cell migration. (**E**) The GPX4 protein expression was detected by Western blotting. (**F**) The ROS content was detected by flow cytometry. (**G**,**H**) MDA and Fe^2+^ contents were detected by using ELISA kits or Iron Colorimetric Assay Kits, respectively. * *p* < 0.01.

To further explore the regulatory role of exosomal GOT1 on ferroptosis, the exosome (150 μL)-incubated PANC-1 cells were transfected with si-GOT1 alone or together with pcDNA-CCR2. We found that CCR2 protein expression was increased in cells incubated with exosomes alone, and the transfection of si-GOT1 reversed the promoting effect of exosomes on CCR2 protein expression, and the transfection of pcDNA-CCR2 reversed the effect of si-GOT1 (Figure 6A). Furthermore, exosome treatment enhanced cell invasion, clonogenicity and migration, but this promotion was reversed by si-GOT1 and transfection with pcDNA-CCR2 again enhanced cell invasion (Figure 6B), clonogenicity (Figure 6C) and migration (Figure 6D). Next, we found that the expression of GPX4 protein was increased (Figure 6E) and the content of ROS (Figure 6F), MDA (Figure 6G) and Fe^2+^ (Figure 6H) were decreased in cells treated with exosomes, suggesting that exosomes inhibit ferroptosis in PANC-1 cells, and this inhibitory effect was reversed after knockdown of GOT1, whereas the overexpression of CCR2 again reversed the promoting effect of si-GOT1 on ferroptosis. Furthermore, SW1990 cells derived from SW1990 cells were transfected with si-GOT1 alone or together with pcDNA-CCR2. It was found that exosomes promoted the expression of CCR2 protein (Supplementary Figure S2A), enhanced cell invasion (Supplementary Figure S2B) and migration (Supplementary Figure S2C), and inhibited ferroptosis (Supplementary Figure S2D–F), and the transfection of si-GOT1 reversed the effect of exosomes, while pcDNA-CCR2 counteracted the effect of si-GOT1 again. The above results indicate that exosomes enriched with GOT1 promote pancreatic cancer cell invasion, clonogenicity, migration and inhibit cellular ferroptosis through upregulated CCR2 expression.

### 3.7. Exosomal GOT1 Inhibited Ferroptosis via Nrf2/HO-1 Axis

**Figure 7:**
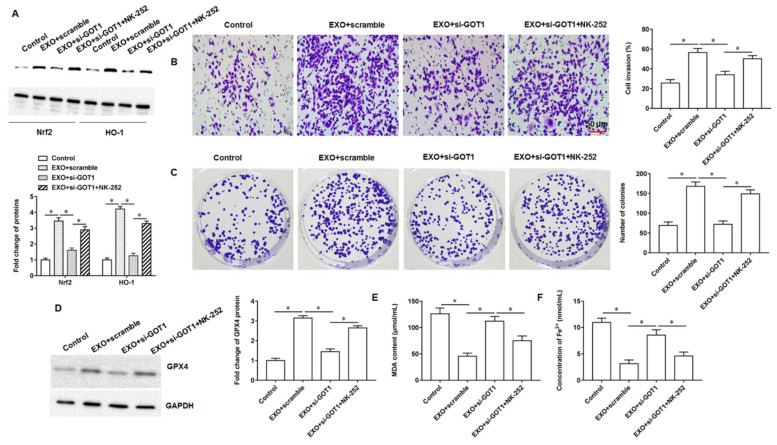
Exosomal GOT1 activates the Nrf2/HO-1 axis. The exosome (150 μL)-incubated PANC-1 cells were transfected with si-GOT1 alone or incubated together with the Nrf2 activator NK-252. (**A**) Western blotting was used to detect Nrf2 and HO-1 protein expression. (**B**) Transwell invasion assay was used to detect cell invasion. (**C**) Cell colony formation assay was used to analyze cell proliferation. (**D**) The GPX4 protein expression was detected by Western blotting. (**E**,**F**) MDA and Fe^2+^ contents were detected by using ELISA kits or Iron Colorimetric Assay Kits, respectively. * *p* < 0.01.

To further explore the specific regulatory mechanism of exosomal GOT1 on ferroptosis, the exosome (150 μL)-incubated PANC-1 cells were transfected with si-GOT1 alone or incubated together with the Nrf2 activator NK-252. We observed that the protein expression of Nrf2 and HO-1 was increased in exosome treated cells, whereas knocking down GOT1 inhibited Nrf2 and HO-1 protein expression, and NK-252 treatment reversed the effect of si-GOT1 (Figure 7A). HO-1, an important source of cellular iron ions, plays a key role in erastin induced cell ferroptosis by inducing the peroxidation of cell membrane lipids, which causes cells to undergo ferroptosis. There have been numerous studies showing that Nrf2 can suppress ferroptosis by increasing the expression of target genes related to iron and ROS metabolism including HO-1 [15,16]. Moreover, exosome treatment enhanced the ability of cell invasion and clonogenicity, and the transfection of si-GOT1 reversed the effect of exosomes, but in the effect of NK-252, the ability of tumor cell invasion (Figure 7B) and clonogenicity (Figure 7C) was again enhanced. Similarly, we observed that knockdown of GOT1 reduced the protein expression of GPX4 (Figure 7D) and increased the contents of MDA (Figure 7E) and Fe^2+^ (Figure 7F) in cells compared with exosome alone treatment, but the promoting effect of si-GOT1 on ferroptosis was reversed under the treatment of NK-252. Moreover, SW1990 cells derived from SW1990 cells were transfected with si-GOT1 alone or incubated together with NK-252. We found that exosomes promoted the expression of Nrf2 and HO-1 proteins (Supplementary Figure S3A), enhanced cell invasion (Supplementary Figure S3B), and inhibited ferroptosis (Supplementary Figure S3C,D), and the transfection of si-GOT1 reversed the effect of exosomes, while NK-252 counteracted the effect of si-GOT1 again. The above results indicate that exosomes enriched with GOT1 promote pancreatic cancer cell invasion, clonogenicity and inhibit cellular ferroptosis by activating the Nrf1/HO-1 pathway.

### 3.8. GOT1 Knockdown Inhibited Tumor Progression

**Figure 8:**
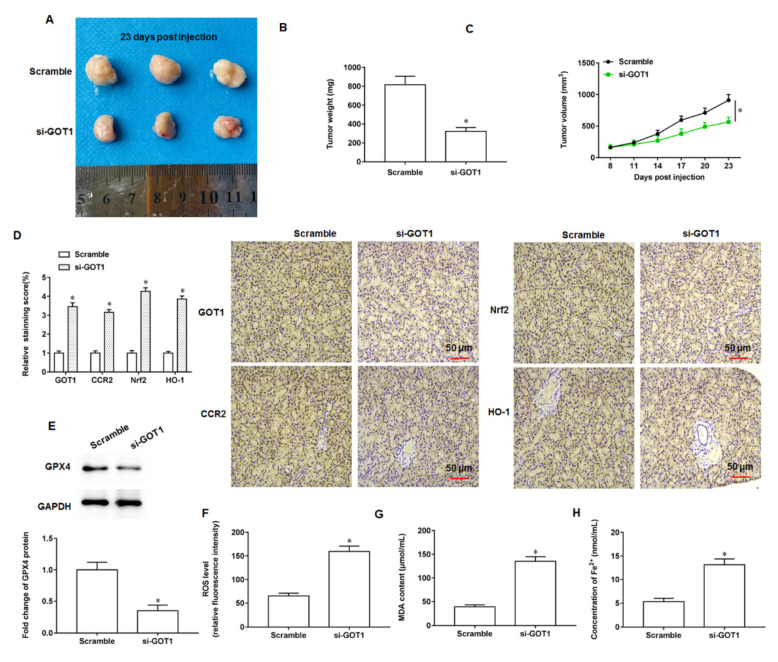
GOT1 knockdown inhibits tumor formation. The exosomes (250 μL) secreted by PANC-1 cells transfected with scramble or si-GOT1, were subcutaneously injected into the armpits of nude mice, and the mice were sacrificed on the day 23 after injection, respectively, and tumor tissues were taken. (**A**) Representative tumor images at day 23 post injection. (**B**) Tumor weight. (**C**) Tumor volume. (**D**) Representative images of immunohistochemical staining of GOT1, CCR2, Nrf2 and HO-1. (**E**) Western blotting was used to detect GPX4 protein expression. (**F**) ROS content was detected by flow cytometry. (**G**,**H**) MDA and Fe^2+^ contents were detected by using ELISA kits or Iron Colorimetric Assay Kits, respectively. * *p* < 0.01.

We observed that tumor formation was inhibited in mice with knockdown of GOT1 expression compared with mice injected with exosomes alone, and representative images were shown in Figure 8A. In addition, we found that both the weight (Figure 8B) and volume (Figure 8C) of tumor tissues from mice with knockdown of GOT1 expression were much smaller than those from control group mice. Next, tumor tissues from both groups of mice were collected and subjected to immunohistochemical histostaining, and we observed a significant decrease in the number of cells positive for GOT1, CCR2, Nrf2 and HO-1 proteins in tumor tissue sections from mice with knockdown of GOT1 expression (Figure 8D). Moreover, Western blotting results suggested that knockdown of GOT1 expression similarly reduced GPX4 protein expression in tumor tissues (Figure 8E). Besides that, the contents of ROS (Figure 8F), MDA (Figure 8G) and Fe^2+^ (Figure 8H) were decreased in the tumor tissues of mice with knockdown of GOT1 expression relative to those in the tumor tissues of control mice. In vivo findings demonstrate that knockdown of GOT1 expression delay tumor progression in nude mice.

## 4. Discussion

The GOT1 gene is located on human chromosome 10 and is approximately 34,955 bp in length. Tumor cell proliferation requires not only the maintenance of intracellular redox homeostasis but also the production of large amounts of RNA, DNA and proteins, and therefore the presence of aspartate must be sufficient. Studies have found that aspartate can be produced not only by Gln in the mitochondrial matrix but also catalytically by GOT1 in the cytoplasmic matrix, so the expression of GOT1 in cells is tightly associated with cell proliferation and the synthesis of aspartate [17]. Oxidative stress is the excessive production of highly reactive molecules such as RNS and ROS in the body, and the degree of oxidation exceeds the scavenging degree of oxides, thus leading to tissue damage. Relative to normal cells, the characteristics of tumor cells are manifested in the increased production rate of reactive oxygen species and the altered redox reaction environment, so that the redox balance inside tumor cells is critical to maintain the survival and proliferation of tumor cells. Tumor cells not only undergo glycolysis to produce NADPH to maintain cellular redox balance, but can also generate NADPH through a non-canonical glutamine pathway mediated by GOT1 [18]. Wiley et al. experimentally demonstrated that aminooxyacetic acid can decrease the NAD+/NADH ratio by inhibiting GOT1, leading to cellular senescence [19]. Another scholar found that ziprasidone could induce glutamine metabolism disorder and redox state imbalance of PDAC cells by targeting GOT1, thereby inhibiting tumor cell proliferation, migration and inducing cell apoptosis [20].

Ferroptosis, a newly discovered mode of programmed death, is a non-apoptotic cell death modality that depends on lipid peroxidation driven processes that require intracellular enrichment of available iron. When cells undergo iron death, a shrunken volume of mitochondria, an increased density of bilayer membranes, and a decrease or disappearance of mitochondrial cristae are clearly observed. Moreover, when iron death occurs, glutathione (GSH) in the cell is depleted, and glutathione peroxidase 4 (GPX4) activity declines, and lipid oxides cannot be metabolized by the GPX4 catalyzed glutathione reduction reaction, followed by the oxidation of lipids by ferrous ions in a manner similar to the Fenton reaction to produce large amounts of reactive oxygen species (ROS), prompting the cell to undergo iron death. Currently, it is found that iron death is polygenically regulated and mainly involves genetic alterations in aspects of iron homeostasis and lipid peroxidative metabolism. Currently, studies have confirmed that GOT1 inhibition promotes pancreatic cancer cell death by triggering ferroptosis [21]. Wang et al. found that miR-9-5p inhibited pancreatic cancer cell proliferation, invasion, glutamine metabolism and redox homeostasis by downregulating GOT1 expression, suggesting that miR-9-5p may serve as a prognostic or therapeutic target in pancreatic cancer [22]. Tumor cells have a high activity of antioxidant system, so tumor cells can have a strong ability to tolerate oxidative stress.

Nuclear factor E2 related factor 2 (Nrf2) is a core transcription factor regulating cellular oxidative stress, which can exert powerful antioxidant/antiapoptotic effects and is an important mechanism of drug resistance in tumor cells. When cells or organisms are exposed to ROS, it can cause the modification of the cytoskeleton associated inhibitory protein Keap1, promoting the dissociation of the keap1-Nrf2 complex, resulting in the translocation of Nrf2 from the cytoplasm into the nucleus. Next, Nrf2 that enters the nucleus can bind to the antioxidant response element, thereby activating the expression of downstream genes that regulate iron and ROS metabolism, including heme oxygenase 1 (HO-1), γ-Glutamylcysteine synthetase and quinone oxidoreductase 1. Yang et al. demonstrated that cetuximab was able to promote RSL3 induced ferroptosis by inhibiting the Nrf2/HO-1 signaling pathway in KRAS mutant colorectal cancer [23]. In bladder cancer progression, erianin is able to promote bladder cancer cell death and cell cycle arrest, and mechanistic studies have shown that this is mediated by accelerating ferroptosis via inducing Nrf2 inactivation [24]. Kuang et al. found that ferroptosis activators promoted mgst1 expression in PDAC cell lines in a Nrf2 dependent manner, and overexpressing mgst1 restored the resistance to ferroptosis in cells with Nrf2 expression knockdown [25].

In this study, we found that GOT1 protein enriched in exosomes secreted by pancreatic cancer cells promoted tumor cell proliferation, invasion and migration and inhibited cell iron death. Furthermore, mechanistic studies revealed that exosomal GOT1 suppressed pancreatic cancer cell iron death and accelerated pancreatic cancer progression by activating the Nrf2/HO-1 axis via upregulation of CCR2 expression, and our study may provide a new reference for pancreatic cancer treatment.

## Data Availability

The datasets used during the present study are available from the corresponding author upon reasonable request.

## Supplementary Materials

The following supporting information can be downloaded at: https://www.mdpi.com/article/10.3390/cells11233893/s1, Figure S1: Effect of exosomal GOT1 (SW1990 cells), Figure S2: Exosomal GOT1 upregulates CCR2 expression (SW1990 cells), Figure S3: Exosomal GOT1 activates the Nrf2/HO-1 axis (SW1990 cells).
