# Supplementary material for: Tumor Cell Derived Exosomal GOT1 Suppresses Tumor Cell Ferroptosis to Accelerate Pancreatic Cancer Progression by Activating Nrf2/HO-1 Axis via Upregulating CCR2 Expression
Source: Cells. 2022 Dec 2;11(23):3893. doi: 10.3390/cells11233893 (PMC9735520; doi:10.3390/cells11233893)
Supplement: Supplementary file 1 [file cells-11-03893-s001.zip › cells-1922034-supplementary.pdf]

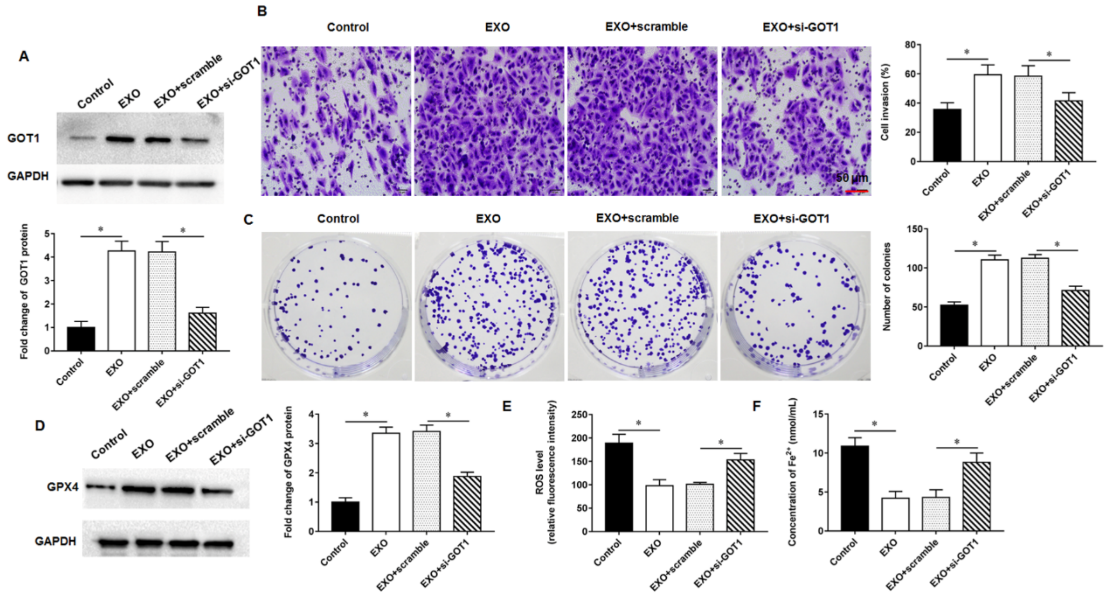

Supplementary Figure S1. Effect of exosomal GOT1 (SW1990 cells). The 150 μL of exosomes were added to SW1990 cells alone or transfected together with si-GOT1. (**A**)**.** Western blotting was used to detect GOT1 protein expression. (**B**)**.** Transwell invasion assay was used to detect cell invasion. (**C**)**.** Cell colony formation assay was used to analyze cell proliferation. (**D**)**.** The GPX4 protein expression was detected by Western blotting. (**E**)**.** The ROS content was detected by flow cytometry. (**F**)**.** The content of Fe^{2+} was detected by using Iron Colorimetric Assay Kits. *P<0.01.

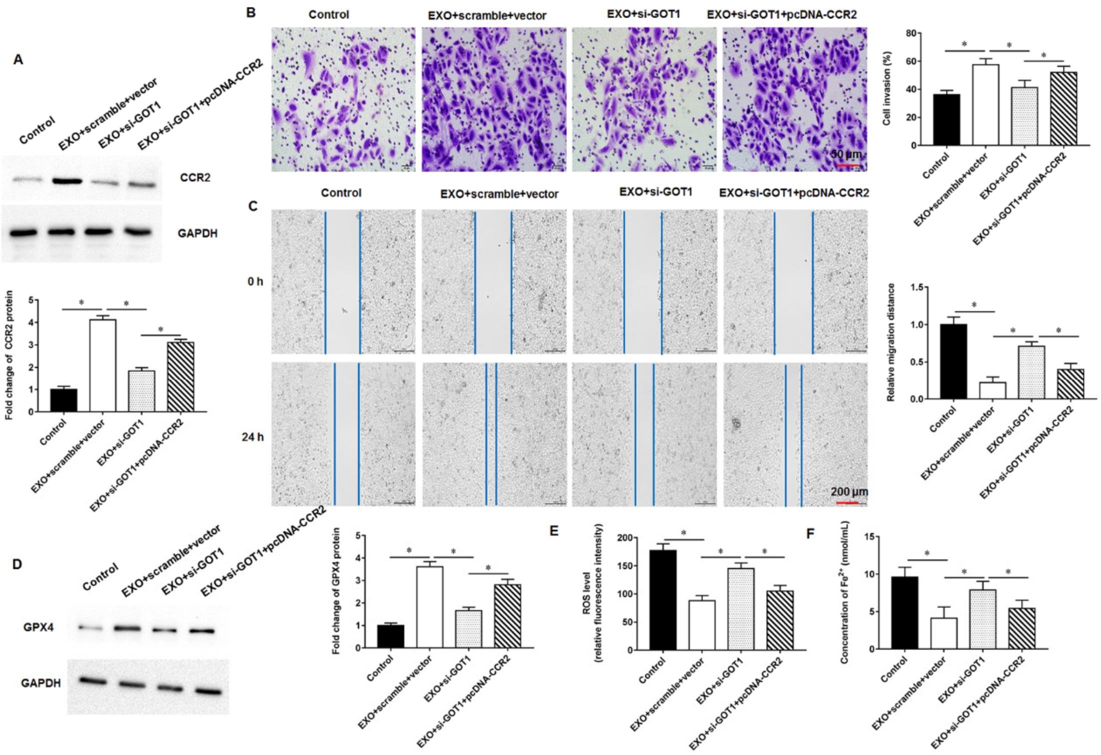

Supplementary Figure S2. Exosomal GOT1 upregulates CCR2 expression (SW1990 cells).

The exosome (150 μL)-incubated SW1990 cells were transfected with si-GOT1 alone or together with pcDNA-CCR2. (**A**)**.** Western blotting was used to detect CCR2 protein expression. (**B**)**.** Transwell invasion assay was used to detect cell invasion. (**C**)**.** Wound healing assay was used to detect cell migration. (**D**)**.** The GPX4 protein expression was detected by Western blotting. (**E**)**.** The ROS content was detected by flow cytometry. (**F**)**.** The content of Fe^{2+} was detected by using Iron Colorimetric Assay Kits. *P<0.01.

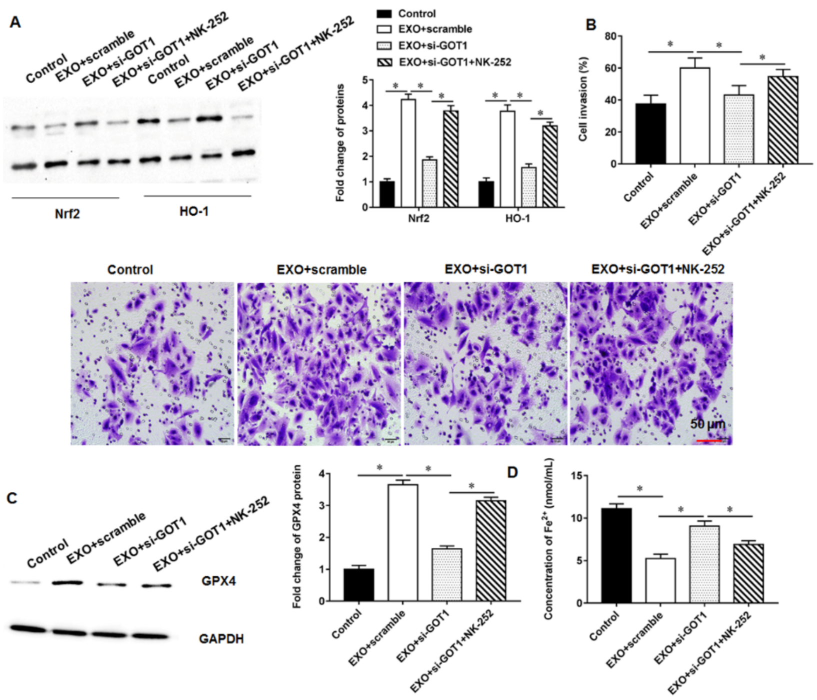

Supplementary Figure S3. Exosomal GOT1 activates the Nrf2/HO-1 axis (SW1990 cells).

The exosome (150 μL)-incubated SW1990 cells were transfected with si-GOT1 alone or incubated together with the Nrf2 activator NK-252. (**A**)**.** Western blotting was used to detect Nrf2 and HO-1 protein expression. (**B**)**.** Transwell invasion assay was used to detect cell invasion. (**C**)**.** The GPX4 protein expression was detected by Western blotting. (**D**)**.** The content of Fe^{2+} was detected by using Iron Colorimetric Assay Kits. *P<0.01.
